# Daidzein Induces Intrinsic Pathway of Apoptosis along with ER α/β Ratio Alteration and ROS Production

**DOI:** 10.31557/APJCP.2021.22.2.603

**Published:** 2021-02

**Authors:** Vinod Kumar, Shyam S Chauhan

**Affiliations:** *Department of Biochemistry, All India Institute of Medical Sciences, New Delhi, India. *

**Keywords:** Daidzein, ER α/β ratio, apoptosis, ROS, Bax and Bcl-2

## Abstract

**Background:**

Low risk of breast cancer is observed among females consuming a moderate quantity of soy throughout their life. The present study was conducted to evaluate the anticancer potential of Daidzein, one of the major Isoflavones in soy using Human breast cancer cells MCF-7.

**Methods:**

MCF-7 were subjected to various doses of Daidzein treatment to determine the IC50 value. Onset of apoptosis was ascertained by AnnexinV assay and caspase 3/7 activity post treatment. Expression of pro-apoptotic protein Bax and anti-apoptotic protein Bcl2 was also assessed to further confirm apoptotic mode of cell death. ROS production post treatment with Daidzein was assessed to ascertain the apoptosis via intrinsic pathway. Expression of *ER α* and *ER β* was evaluated by western blot analysis.

**Results:**

Human breast cancer cells MCF-7 were found to be sensitive to Daidzein treatment, with an IC_50_ value of 50µM. Increased percentage of treated cells stained with Annexin V confirmed apoptosis mediated cell death. Activity of Caspase 3/7 activity was found to be 1.4-fold higher in Daidzein treated cells than control cells, confirming apoptosis. Daidzein caused over expression of Bax and down-regulated expression of* Bcl2*. There has been an outburst of ROS in Daidzein treated cells indicating that Daidzein induces apoptosis via intrinsic pathway. A decrease in the expression of *ER α* and increase in levels of *ER β* has been observed which are conducive indicator of apoptosis.

**Conclusions:**

In conclusion, the present study suggests that Daidzein induces apoptosis in MCF-7 cells by mitochondrial pathway along with lowering the ratio of ER α/β and an outburst of Reactive Oxygen Species(ROS).

## Introduction

Breast cancer, along with lung and bronchial cancer, has been estimated to account for about half of cancer cases in women. Of these, breast cancer alone contributes for 29% cases in females (Siegel et al., 2016). Prevalence of breast cancer is higher in European and North American countries than Asian countries. Interestingly, breast cancer incidences in Asians have increased and eventually the data has become at par with Europeans and North Americans on migration to these countries (Jemal et al., 2011). Dietary factors have been suggested to be one of the major causes along with environmental and life style changes (Deapen et al., 2002). One of the striking differences observed between the diets of Western and Asian populations is consumption of biologically active phenolic compounds, phytoestrogen enriched foods especially soy and soy based food in Asian countries (Limer and Speirs, 2004; Shu et al., 2009). In traditional Asian diets, an estimated 20-30 gram of soy protein per day is consumed, which contains about 100mg of isoflavones as against Western diets which contain less than 1gram of soy protein (Xiao, 2008).

Phytoestrogens are non-steroidal compounds derived from plants or from the in vivo metabolism of precursors in our diet. More than 300 plants are known to contain phytoestrogens (Farnsworth et al., 1975), with soy and flax considered the richest sources. What makes soy unique is the presence of Isoflavones, bioactive compounds which have quite similar chemical structure to Estradiol. The three Isoflavones constituents in soy are Genistein, Daidzein and Glycitein. Isoflavones share many properties of endogenous estrogens and, thus, compete with endogenous estrogens by binding to estrogen receptors and mimicking estrogen messages to the cells (Kurzer and Xu, 1997).

Isoflavones exert their effects through interaction with Estrogen Receptors α and β with higher binding affinity to Estrogen Receptor β (ERβ) as compared to Estrogen Receptor α (ERα) (Pons et al., 2016). ERα is known to promote cell growth and differentiation. On the other hand, ERβ is relevant in cytostatic and differentiation activities and counters the proliferative effects of ERα in mammary cancer cells growth (Chang et al., 2006; Rajah et al., 2009; Sotoca et al., 2008). This indicates that the differential expression of ERs α and β determines the induction or inhibition of apoptosis. An increased ratio of ER α/β will lead to cell proliferation whereas a lowered ratio of the same will lead to apoptosis. Amongst the mechanisms of actions, some anti-cancer treatments lead to the generation of Reactive Oxygen Species (ROS) producing oxidative stress in cancer cells leading to their death (Pons et al., 2016). 

Herein the molecule taken for study, Daidzein, an isoflavanoid has been reported to be a non-toxic compound with ability to inhibit the growth of tumors of pancreatic (Guo et al., 2004a), colon (Guo et al., 2004b) and ovarian (Gercel-Taylor et al., 2004) cancers. Daidzein induce cells death in BEL7402 hepatoma cells by mitochondrial apoptosis (Han et al., 2015) and suppression of matrix metalloproteinase (MMP) 2 while inhibiting invasion by MDA-MB-231 cells invasion in breast cancer cells (Magee et al., 2014). Hirota et al., (2010) have observed the mitochondrial apoptosis during arrest of growth differentiation of human pre-adipocytes, AML-I by Daidzein (Hirota et al., 2010). 

In this study anticancer potential of Daidzein has been assessed by cytotoxic activity and apoptotic induction in MCF-7 cells. We further explored the mechanistic pathway of Daidzein by measuring the expression levels of Estrogen Receptors α and β, Bax and Bcl-2 by Western blotting. 

No studies have been carried out to define the manner in which the Daidzein molecule binds to different proteins of this pathway. So in this context, it is really difficult to understand the putative structural and functional role of Daidzein molecule inside the active site pocket in ER-α/β, BCL2 and BAX protein. To accomplish this and as described in this manuscript, we have performed different types of molecular docking experiments to investigating the binding energy and stability of protein- Daidzein complexes with above said proteins. Our study might be further explored by structural biologists to investigate the geometrical and electronic positions of Daidzein molecule on electron density maps.

## Materials and Methods

*Reagents*

MCF-7 Cells were procured from National Centre for Cell Science (NCCS), Pune, India. Phenol free and Phenol containing Dulbecco’s Modified Eagle’s medium (DMEM) high glucose and Fetal Bovine Serum (FBS) were purchased from GIBCO (Paisley, UK). Antibodies against ER α, ERβ, Bax, Bcl2 and α–tubulin were purchased from AbCam (Cambridge, MA, USA). Secondary antibodies,2′,7′-Dichlorofluorescin diacetate (DCFDA,Catalogue number D6883) for ROS detection and Daidzein were bought from Sigma–Aldrich (St. Louis, MO, USA). Annexin V-Fluorescein Isothiocynate (FITC) detection kit was purchased from BD Biosciences (San Jose, CA,USA)Cat No. 556547. Caspase3/7 detection kit was procured from Promega (Madison,WI, USA) Caspase-Glo^®^ 3/7 Assay; Catalogue number G8090.Routine chemicals used were obtained from Sigma–Aldrich (St. Louis, MO, USA), Bio-Rad Laboratories (Hercules, CA, USA).

*Maintenance and treatment of cells*

MCF-7 cells were treated in phenol-free DMEM medium supplemented with10% charcoal-stripped Fetal Bovine Serum (FBS) (Hyclone), antibiotics (100 U/ml penicillin, and 100 µg/ml streptomycin) and 2 mM L-glutamine for 48 h at the beginning of each experiment. The cells were kept in an exponential growth phase during experiments.

*Cell Viability assay*

Cell growth inhibition using Daidzein was performed using the 3-(4, 5-dimethylthiazol-2-yl)-2,5-diphenyltetrazolium bromide (MTT) assay. Briefly, 5×10^3^ MCF-7 cells were seeded in 96-well plates and treated with various concentrations of Daidzein viz. 0, 25, 50 and 100µM was given for 24,48 and 72 hrs. After treatment, 20 μl MTT (5 mg/ml) was added and incubated for 4 hours at 37^o^C. Now, 100 µl of DMSO was added to each well to dissolve the resulting formazan crystals. Absorbance was measured at 490 nm in and ELISA reader. Percentage of Daidzein-induced cell growth inhibition was calculated by comparison to DMSO-treated control cells.

*Cytometry analysis for study of Daidzein induced apoptosis using Annexin V staining*

1X105 MCT-7 cells were seeded in 6 well culture plate in phenol free DMEM supplemented with 10% charcoal stripped FBS and were allowed to adhere overnight. The cells were then allowed to synchronize for 6 hrs in DMEM supplemented with 1%FBS. After that, media in test flask was replaced with that containing IC_50_ dose i.e. 50µM Diadzein in DMSO and that of control was replaced by media containing equal amount of DMSO and incubated for 48 hrs. After 48 hrs, cells were harvested and prepared for Annexin V staining by FACS as per manufacturer’s protocol (Annexin V FITC Apop Dtec Kit I, Catalogue No. 556547 BD Biosciences). Briefly, 5 µl each of Annexin V–FITC PI were added to cells’ pellet and vortexed gently. Then the cells were incubated for 30 minutes at room temperature in dark and 400µl of binding buffer was added to each tube. Finally, cells were analyzed by flow cytometer (Becton Dickinson; Ex, 488 nm and Em, 530 nm).

*Study of level of Caspase3/7 in Diadzein treated cells*

5X10^3 ^MCT-7 cells were seeded in 96 well plate and allowed to adhere overnight. Next day, media in test well was replaced with that containing 50µMolar diadzein in DMSO. That of control was replaced by media containing equal amount of DMSO. Cells were allowed to grow for 48 hrs. After 48 hrs, equal amount of Caspase3 detection solution (Promega Caspase-Glo^®^ 3/7 Assay, Catalogue numberG8090) was overlaid and allowed to incubate for 3 hrs. Caspase3/7 level was determined by taking readings in luminometer. 

*Apoptotic markers and ER α and ERβ analysis by Western Blotting*

5×10^5^ MCF-7 cells were seeded in 25Cm^2^ flask in phenol free DMEM supplemented with 10% charcoal-stripped FBS and were allowed to adhere overnight. The cells were then allowed to synchronize for 6 hrs in DMEM supplemented with 1% charcoal-stripped FBS. Media in test flask was replaced with that containing 50µM Diadzein in DMSO and incubated for 48 hrs. After 48 hrs, cells were harvested and lysates prepared in RIPA lysis buffer. Western blotting was done after protein estimation using BCA and using corresponding antibodies. 60 µg protein from each control was resolved on polyacrylamide gel (SDS PAGE) (12.5%). These resolved proteins were transferred to polyvinylidene difluoride (PVDF) membrane and the membrane was blocked with 3% bovine serum albumin (BSA) in TBST for one hour at room temperature. Membrane was probed with primary antibodies at RT (25^o^C) for 1 hour or at 4oC overnight. Each of the targeted proteins was immune probed using specific antibodies (Bax, Bcl-2, ER α, ERβ, and α-tubulin).

The membrane was washed three times with TBST and then incubated for 1 hour at RT with Horseradish Peroxidase (HRP) conjugated secondary antibody. After three washes of 10 minutes each with TBST, membrane was subjected to Chemiluminol treatment and analyzed in auto chemiluminescence. 

*Reactive Oxygen Species measurement*

Reactive oxygen species (ROS) were measured on the basis of the intracellular peroxide-dependent oxidation of 2’,7’–dichlorofluorescin diacetate (DCFDA)to form the fluorescent compound dichlorofluorescein (DCF). 5×10^3 ^MCF-7Cells per well were seeded on to a 96-well plate and cultured for 24 h. After washing twice with PBS, fresh medium containing 0,50 and 100 µM Daidzein was added and cells were incubated for various time intervals viz. 0 ,30 and 60 minutes.0.03% H_2_O_2_ was used as a positive control. Then, 20 µM DCFDA in DMSO was added and cells were incubated for 30 min at 37oC. The cells were washed twice with PBS, 100 µM of PBS were added to each well and fluorescence intensity was measured using FLUOstar Omega Filter-based multi-mode micro plate reader, excitation at 490nm and emission at 525nm.

*Computational molecular docking study*

The atomic coordinates of four crystal structures from human Estrogen Receptor α, Estrogen Receptor β, Bcl2 and Bax proteins were obtained from Protein Data Bank (Berman et al., 2002) for investigation of molecular docking. The PDB structure of 5UFX (Fanning et al., 2018) from Estrogen Receptor α, 5TOA (Souza et al., 2017) from Estrogen Receptor β, 5JSN (Berger et al., 2016) from Bcl2 and 5W6O (Kall et al., 2018) from Bax protein have been included in the present study and were taken as receptor. These crystal structures (for breast cancer proteins) have been solved in different space groups, pH and with different resolutions. The A molecule from each structure has been separated from their respective X-ray structures (excluding water molecules and legends) using Swiss-PDB viewer program (Guex and Peitsch, 1997) for further docking study with ligand Daidzein. The structure of Daidzein molecule was obtained from protein databank for further molecular docking study to four respective proteins for investigation their binding affinity with ligand. There have been a series of molecular docking programs to be reported, but different molecular docking programs may be suitable for specific drug targets. We have employed AutoDock Vina (version 1.1.2) (Trott and Olson, 2010) and Blind docking program (Sánchez-Linares et al., 2012) for computational docking study of Daidzein molecule with four proteins. Required PDBQT files of the four receptor proteins were generated using AutoDock Tools v.1.5.4 by assigning Kollman united atom charges (Weiner et al., 1984). The structures for ligand were converted into PDBQT file after including their partial atomic charges using Gasteiger method (Gasteiger and Marsili, 1980). Grid point spacing was set at 1 Å and different grid points were taken in each direction. As the location of ligand inside the protein (His524 and Glu353 of estrogen receptor α, Glu305 and His475 of estrogen receptor β, Arg146 and Asp140 of BCL2 and Glu61, Lys64 and Asp68 of BAX protein) was already known, so grid box was centered near the proper binding site of each protein. Vina automatically calculated the grid map for searching. All other docking parameters were assigned to their default values. We took the 10 best results (of docked complexes) according to their binding affinity. For further investigation to binding affinity of Daidzein molecule with four receptors, we have also conducted blind docking study using Blind docking program.

*Statistical calculations*

Each experiment was repeated three times and statistical analysis was carried out using one-way analysis of variance tests with the Graph Pad Prism Version 6.04 system. Data are presented as mean values +/-standard deviation. P value of<0.05 has been considered statistically significant.

## Results

*Cell Viability Assay*

The treatment of MCF-7 cells with control, 25, 50, and 100µM of Daidzein showed a dose and time-dependent cell growth inhibition. The inhibition of growth rate is by 5%,15% and 24%, respectively, at 24 h, compared with control (P =0.047); 8,20 and 27%, respectively, at 48 h (P =0.047), and 19,27 and 42%, respectively, at 72 h (P =0.047), all values compared to untreated cells. 

*Confirmation of apoptosis by flow cytometry*

Flow cytometry analysis showed substantial change in profile of cells after treatment with 50 μm of Daidzein. Cells stained with Annexin V FITC and PI were classified as necrotic (Quadrant1; Annexin−/PI+), late apoptotic (Quadrant2; Annexin+/PI+), intact cells (Quadrant4; Annexin−/PI−) and early apoptotic cells (Quadrant3; Annexin+/PI−) as explained in figure2(a) and 2(b). The results show quantitatively induction of apoptosis by Daidzein in MCF-7 cells. There is an increase in percentage of cells undergoing early and late apoptosis in Daidzein treated cells compared to control cells.

*Enhanced activity of Caspase3/7 in Daidzein treated cells*

There was an enhancement by 1.4-fold in the activity of Caspase3/7 enzyme in Daidzein treated MCF-7 cells compared to those in untreated ones, Figure 3. This confirms the apoptotic cell death in treated cells. The experiment was repeated three times and average of luminescence in treated versus control cells is plotted.

*Western blot analysis*

Treatment of MCF-7 cells with 50μm Daidzein lead to a significant increase in Bax expression. However it resulted in significant decrease in *Bcl-2* expression, Figure 4 .Overall, there was an increase in Bax/Bcl-2 ratio in Daidzein treated cells compared to control cells which is a driving force for intrinsic pathway of apoptosis induction, Figure 4a.Simultaneously, there was under expression of ER α, a pro inflammatory factor and enhanced expression of *ER β*, anti-inflammatory factor in Daidzein treated cells as against control cells; altering the ratio of ER α/β in favour of growth suppression, Figure 4c.

*Reactive Oxygen Species (ROS) analysis*

Outburst of ROS was observed after treatment with0, 50 and 100 µM of Daidzein (Figure 5). There is no change in emission values in control. In case of Daidzein treatment, emission is more with time (P <0.001) indicating production of ROS.

*Computational docking analysis*

The binding free energy obtained from Auto Dock program of Daidzein molecule is -9.30 kcal/mol for Estrogen Receptor α, -8.30 kcal/mol for Estrogen Receptor β, -6.90 kcal/mol for BCL2, -5.90 kcal/mol for Bax protein. In addition, binding free energy which was obtained from Blind docking program of Daidzein molecule is -9.30kcal/mol for Estrogen Receptor α, -8.10 kcal/mol for Estrogen Receptor β, -6.70 kcal/mol for Bcl2, -5.70 kcal/mol for Bax protein.

**Figure 1:**
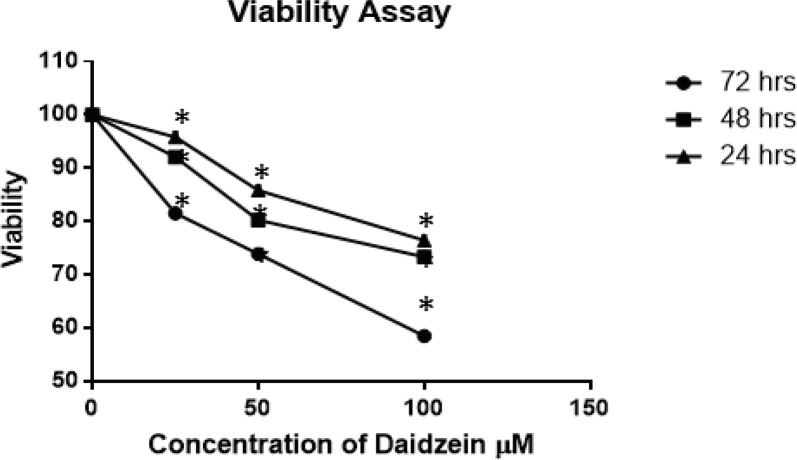
MCF-7 Cells Viability Analysis after Daidzein Treatment. MCF-7 cells were treated with varying concentrations of daidzein for 24, 48 and 72 h. After treatment, 20 μl of 5 mg/ml MTT was added and incubated for 4 hours at 37o C. After this, 100 µl of DMSO was added to each well to dissolve the resulting formazan crystals. Absorbance was measured at 490 nm in and ELISA reader. Data are presented as means of standard deviation from three independent experiments. Each experiment was conducted in triplicate (P = 0.047).

**Figure 2:**
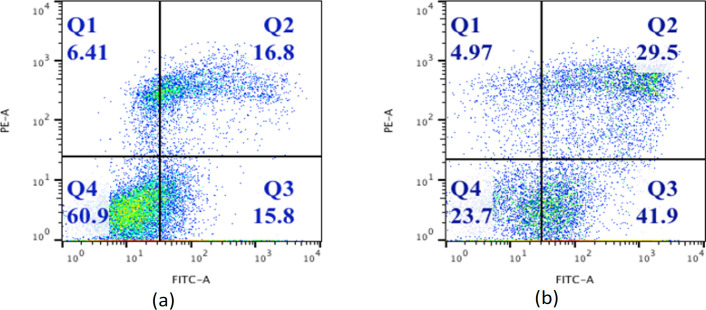
Daidzein Induced Apoptosis in MCF-7 Cells. 1×10^5^ MCT-7 cells were treated with 50µM Diadzein for 48 hrs. and analysed for apoptosis by Annexin V assay by flow cytometry. After 48 hrs. 5 µl each of Annexin V and FITC PI were added to cells’ pellet and vortexed gently. Then the cells were incubated for 30 minutes at room temperature in dark and 400µl of binding buffer was added to each tube and subjected to analysis by flow cytometer (Becton Dickinson; Ex, 488 nm and Em, 530 nm). Flow cytometry analysis of MCF-7 cells shows apoptotic cells stained for Annexin V and Propidium Iodide (PI) after treatment with 50µM daidzein (panel b) as compared to untreated cells (panel a). In both the panels, Quadrant1; Annexin−/PI+ (necrosis), Quadrant2; Annexin+/PI+(late apoptotic), Quadrant3; Annexin+/PI−(early apoptotic cells) and Quadrant4; Annexin−/PI− (intact cells). There is an increase in apoptosis in daidzein treated cells as compared to untreated ones from 16.8% to 29.5%. Also, there is substantial increase in number of early apoptotic cells in treated cells ;15.8% to 41.9% as against control cells

**Figure 3:**
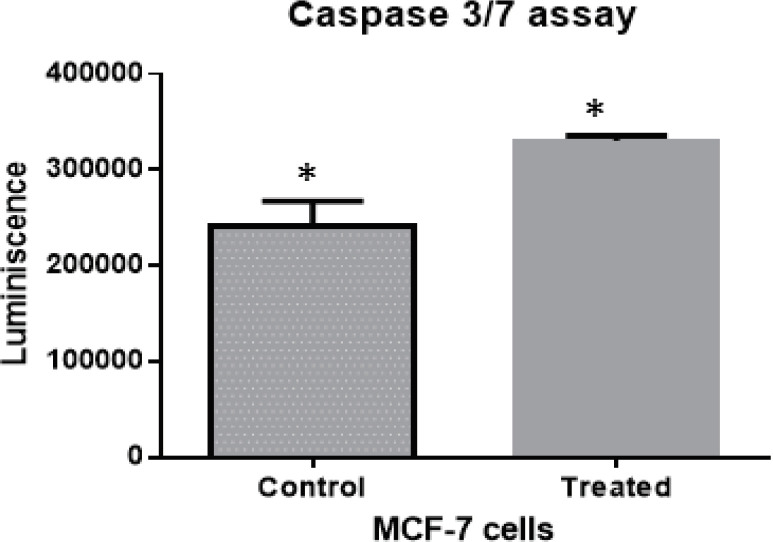
Activity of Apoptotic Marker Caspase 3 /7 has been Observed to be more in Diadzein Treated MCF-7 Cells as Compared to Control Cells. There is an increase of 1.4 times in the activity of caspase3/7 in daidzein treated cell lines as compared to untreated ones. The data represent plot of thrice repeated analysis with a p value of 0.0194

**Figure 4:**
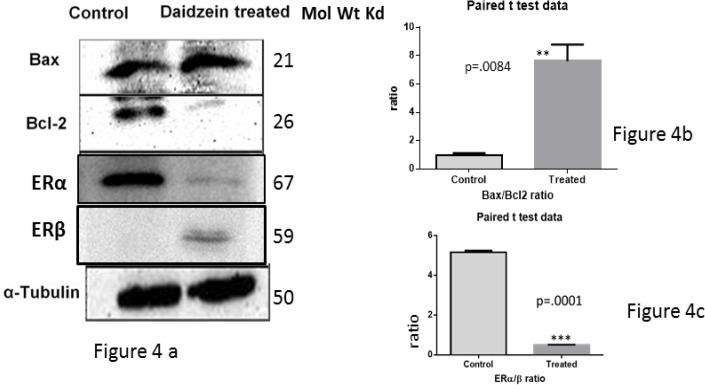
Apoptotic Markers were Analyzed by Western Blotting of Lysates of MCF-7 Cells after Treatment with 50 μm Daidzein against Control Cells. 60 μg of protein was loaded for each sample. Representative blots for Bax, Bcl-2 , ER α, ERβ and α-tubulin show the expression profile of concerned markers (4a). α-tubulin has been used as an internal loading control. Densitometric analysis of Bax vs Bcl-2 (4b) shows and increase in the ratio of Bax/Bcl-2 by a factor of about ten times (p=.0084) and that of ER αand ER β (4c) shows a decrease in the ratio of ER α /ER β by about 6 times (p=.001) . These observations collectively indicate inhibition of growth of cells and induction of apoptosis by intrinsic pathway of apoptosis

**Figure 5:**
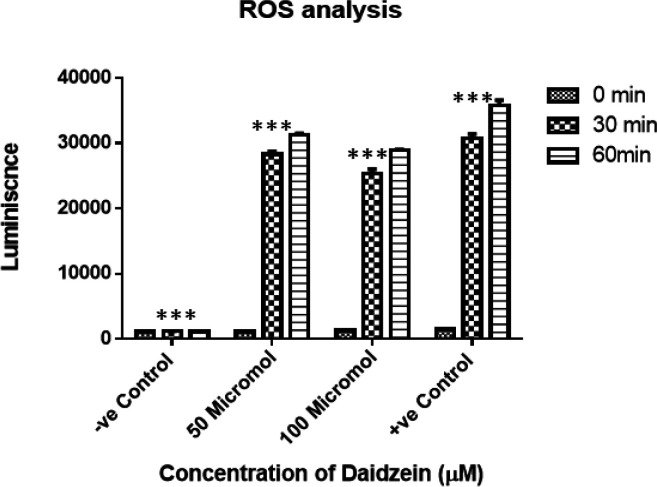
Generation of Reactive Oxygen Species (ROS) in 50μm and 100 μm Daidzein Treated MCF-7 Cells. There is an outburst of ROS in 50 and 100 μm Daidzein treated cells as against control cells. As can be seen, the ROS emission is higher in 50 μm Daidzein treated cells in both the time frames, this is justified as the standard inhibitory concentration of daidzein which is capable of inducing apoptosis. Luminescence is higher in +ve control (0.03% H_2_O_2_). The data is statistically significant in all the four subsets of observation with p value in each case being 0.001

## Discussion

This study was focused on exploring the mechanism of soy isoflavone Daidzein in induction of cell death in breast cancer cells MCF-7. Herein, we have explored the events upstream of apoptosis induction and control of cell growth by Daidzein in MCF-7 cells. We observed an increase in expression of pro-apoptotic factor Bax with concomitant decrease in expression of anti-apoptotic factor Bcl-2. Increased production of ROS post treatment corroborates the mitochondrial involvement in cell killing.

It is well-established that soy Isoflavones Genistein, Daidzein and Glycitein cause inhibition of cell growth by various mechanisms including regulation of apoptosis, cell proliferation and survival and inhibition of angiogenesis. Along with above said mechanism, various actions of isoflavones are accomplished by interaction with estrogen receptors alpha (ERα) and beta (ERβ) (Rahman, 1988). Both *ERα* and *ERβ* are encoded by distinct genes; *ESR1 *and *ESR2* respectively and are members of the steroid receptor super family. ERα is associated with aberrant proliferation, inflammation and development of malignancy whereas *ERβ* opposes ERα mediated cell proliferation by modulating the expression of many *ERα*-regulated genes and exhibiting anti-migratory and anti-invasive properties in cancer cells (Thomas and Gustafsson, 2011). Isoflavones interact with receptors by binding to ligand binding domains of ERs. It further binds to sequence-specific response elements known as estrogen response elements from DNA and the transcription of target gene is then triggered. Also, *ERα* and *ERβ* can regulate gene transcription by rapidly activating Src/mitogen-activated protein (Src/MAP) kinase (Migliaccio et al.,1996) phosphatidylinositide 3-kinases/Akt (PI3K/Akt (Castoria et al., 2001) and other direct DNA-binding transcription factors, such as activating protein 1 (AP1), specificity protein 1 (SP1), cAMP response element-binding protein (CREB), nuclear factor-κB (NF-κB). Naturally occurring 17-β-estradiol has a higher affinity for ERβ compared to that for ERα. The soy analogues have similar trend in affinity for receptors but of lower magnitude compared to natural one. Daidzein binds to ERβ more preferentially as compared to ERα (Kuiper et al., 1998). In our study; we have observed a decline of about 50 percent in expression of *ERα* and an increase of about four times for ERβ in Daidzein treated cells. This indicates that Daidzein induces cell growth inhibition in MCF-7 cells by lowering the ratio of ERα /ERβ by approximately ten times. This observation is in consonance with the earlier established fact that the ERα/ERβ ratio is tilted upward during tumorigenesis due to up regulation of ERα mRNA levels within the tumor compartment (Leygue et al., 1998).

We further explored that Daidzein mediates cell death by intrinsic apoptotic pathway involving alteration of mitochondrial membrane potential due to enhanced Bax/Bcl2 ratio resulting in Cytochrome C release in to cytosol and activation of Caspase 9. Activation of Caspase 9 by Daidzein (Choi and Kim, 2008), indicates activation of apoptosis by Daidzein through intrinsic pathway preferentially over extrinsic one.

The onset of apoptosis was confirmed by Annexin V assay by flow cytometry. We have observed a significant increase in cells stained with Annexin V after treatment with 50µM Daidzein indicating initiation of apoptosis. 

Soy isoflavones have been long associated with modulation of the oxidative stress in breast cancer cells by favoring ROS accumulation or by decreasing the antioxidant defense which ultimately leads to cell death. In ER–positive breast cancer cells like MCF-7, higher ROS levels and DNA damage are induced by estrogen through ER mediated mechanisms. In nutshell, the ERα/ERβ ratio is a strong determinant of oxidative status in response to estrogen leading to heightened oxidative damage along with low levels of antioxidant enzymes. We have observed an outburst of ROS in diadzein treated cells in time and dose dependent pattern. This indicates an enhanced damage due to oxidative stress in the cells post treatment with isoflavone.

We have further confirmed the induction of apoptosis by intrinsic pathway of apoptosis by Bax and Bcl2 profiling in control versus treated cells. Increased ratio of Bax/Bcl2 in Diadzein treated cells was observed as compared to control by a magnitude of ten. Enhanced ratio of Bax/Bcl2 is known to mediate permeabilization of mitochondrial membrane and release of Cytochrome C in to cytosol which activates pro caspase 3/7 resulting in apoptotic cell death. Based on this, we further confirmed the activation of Caspase3/7 by analyzing the levels of Caspase 3/7 in Daidzein treated cells which was found to be enhanced in treated cells by a magnitude of about 1.4.

The focus of our computational study was emphasized on binding study of Daidzein molecule with four protein structure to characterize their functional roles in protein stability. Our computational docking study clearly indicated that estrogen receptor α is highly inhibited than estrogen receptor β, so expression level ER- α is less than ER-β and the ratio of ER- α and β is decreased by six times. Similarly, the Bcl2 protein is highly inhibited than Bax protein thus the expression of Bcl2 is less than Bax protein and their ration of Bax to Bcl2 is increased by 10 times. The results from this computational study could be of interest to experimental community for structural biology and provide a testable hypothesis for experimental validation.

In conclusion, this study concludes that soy isoflavone Daidzein induces cell death in breast cancer cells MCF-7 mediated by over-expression of pro-apoptotic protein Bax and lowering the expression of anti-apoptotic marker Bcl2. This altered ratio of Bax/Bacl2 leads to ROS outburst and release of Cytochrome C due to change in mitochondrial membrane permeability. The molecular modeling and docking approach also supported the experimental results. Further elaborated mechanistic studies confirmed that along with induction of intrinsic pathway of apoptosis, Diadzein also changes the ratio of Estrogen Receptors α and β and tilts the balance in favour of suppression of cell proliferation. 

## References

[B1] Berger S, Procko E, Margineantu D (2016). Computationally designed high specificity inhibitors delineate the roles of BCL2 family proteins in cancer. eLife.

[B2] Berman HM, Battistuz T, Bhat TN (2002). The protein data bank. Acta Crystallogr D Biol Crystallogr.

[B3] Castoria G, Migliaccio A, Bilancio A (2001). PI3-kinase in concert with Src promotes the S-phase entry of oestradiol-stimulated MCF-7 cells. EMBO J.

[B4] Chang EC, Frasor J, Komm B, Katzenellenbogen BS (2006). Impact of estrogen receptor beta on gene networks regulated by estrogen receptor alpha in breast cancer cells. Endocrinology.

[B5] Choi EJ, Kim G-H (2008). Daidzein causes cell cycle arrest at the G1 and G2/M phases in human breast cancer MCF-7 and MDA-MB-453 cells. Phytomedicine Int J Phytother Phytopharm.

[B6] Deapen D, Liu L, Perkins C (2002). Rapidly rising breast cancer incidence rates among Asian-American women. Int J Cancer.

[B7] Fanning SW, Hodges-Gallagher L, Myles DC (2018). Specific stereochemistry of OP-1074 disrupts estrogen receptor alpha helix 12 and confers pure antiestrogenic activity. Nat Commun.

[B8] Farnsworth NR, Bingel AS, Cordell GA (1975). Potential value of plants as sources of new antifertility agents II. J Pharm Sci.

[B9] Gasteiger J, Marsili M (1980). Iterative partial equalization of orbital electronegativity—a rapid access to atomic charges. Tetrahedron.

[B10] Gercel-Taylor C, Feitelson AK, Taylor DD (2004). Inhibitory effect of genistein and daidzein on ovarian cancer cell growth. Anticancer Res.

[B11] Guex N, Peitsch MC (1997). SWISS-MODEL and the Swiss-PdbViewer: an environment for comparative protein modeling. Electrophoresis.

[B12] Guo J-M, Xiao B-X, Dai D-J (2004). Effects of daidzein on estrogen-receptor-positive and negative pancreatic cancer cells in vitro. World J Gastroenterol.

[B13] Guo JM, Xiao BX, Liu DH (2004). Biphasic effect of daidzein on cell growth of human colon cancer cells. Food Chem Toxicol Int J Publ Br Ind Biol Res Assoc.

[B14] Han B-J, Li W, Jiang G-B (2015). Effects of daidzein in regards to cytotoxicity in vitro, apoptosis, reactive oxygen species level, cell cycle arrest and the expression of caspase and Bcl-2 family proteins. Oncol Rep.

[B15] Hirota K, Morikawa K, Hanada H (2010). Effect of genistein and daidzein on the proliferation and differentiation of human preadipocyte cell line. J Agric Food Chem.

[B16] Jemal A, Bray F, Center MM (2011). Global cancer statistics. CA Cancer J Clin.

[B17] Kall SL, Delikatny EJ, Lavie A (2018). Identification of a unique inhibitor-binding site on choline kinase α. Biochemistry.

[B18] Kuiper GG, Lemmen JG, Carlsson B (1998). Interaction of estrogenic chemicals and phytoestrogens with estrogen receptor beta. Endocrinology.

[B19] Kurzer MS, Xu X (1997). Dietary phytoestrogens. Annu Rev Nutr.

[B20] Leygue E, Dotzlaw H, Watson PH, Murphy LC (1998). Altered estrogen receptor alpha and beta messenger RNA expression during human breast tumorigenesis. Cancer Res.

[B21] Limer JL, Speirs V (2004). Phyto-oestrogens and breast cancer chemoprevention. Breast Cancer Res BCR.

[B22] Magee PJ, Allsopp P, Samaletdin A, Rowland IR (2014). Daidzein, R-(+)equol and S-(-)equol inhibit the invasion of MDA-MB-231 breast cancer cells potentially via the down-regulation of matrix metalloproteinase-2. Eur J Nutr.

[B23] Migliaccio A, Di Domenico M, Castoria G (1996). Tyrosine kinase/p21ras/MAP-kinase pathway activation by estradiol-receptor complex in MCF-7 cells. EMBO J.

[B24] Pons DG, Nadal-Serrano M, Torrens-Mas M (2016). The phytoestrogen genistein affects breast cancer cells treatment depending on the ERα/ERβ Ratio. J Cell Biochem.

[B26] Rajah TT, Du N, Drews N, Cohn R (2009). Genistein in the presence of 17beta-estradiol inhibits proliferation of ERbeta breast cancer cells. Pharmacology.

[B27] Sánchez-Linares I, Pérez-Sánchez H, Cecilia JM, García JM (2012). High-throughput parallel blind virtual screening using BINDSURF. BMC Bioinformatics.

[B28] Shu XO, Zheng Y, Cai H (2009). Soy food intake and breast cancer survival. JAMA.

[B29] Siegel RL, Miller KD, Jemal A (2016). Cancer statistics, 2016. CA Cancer J Clin.

[B30] Sotoca AM, Ratman D, van der Saag P (2008). Phytoestrogen-mediated inhibition of proliferation of the human T47D breast cancer cells depends on the ERalpha/ERbeta ratio. J Steroid Biochem Mol Biol.

[B31] Souza PCT, Textor LC, Melo DC (2017). An alternative conformation of ERβ bound to estradiol reveals H12 in a stable antagonist position. Sci Rep.

[B32] Thomas C, Gustafsson J-Å (2011). The different roles of ER subtypes in cancer biology and therapy. Nat Rev Cancer.

[B33] Trott O, Olson AJ (2010). AutoDock Vina: improving the speed and accuracy of docking with a new scoring function, efficient optimization, and multithreading. J Comput Chem.

[B34] Weiner SJ, Kollman PA, Case DA (1984). A new force field for molecular mechanical simulation of nucleic acids and proteins. J Am Chem Soc.

[B35] Xiao CW (2008). Health effects of soy protein and isoflavones in humans. J Nutr.

